# Combined Genetic and Transcriptional Study Unveils the Role of *DGAT1* Gene Mutations in Congenital Diarrhea

**DOI:** 10.3390/biomedicines13081897

**Published:** 2025-08-04

**Authors:** Jingqing Zeng, Jing Ma, Lan Wang, Zhaohui Deng, Ruen Yao

**Affiliations:** 1 Digestive Department of Pediatrics, Shanghai Children’s Medical Center, School of Medicine, Shanghai Jiao Tong University, 1678 Dongfang Road, Shanghai 200127, China; zengjingqing@scmc.com.cn (J.Z.); wanglan@scmc.com.cn (L.W.); 2 Department of Pathology, Shanghai Children’s Medical Center, School of Medicine, Shanghai Jiao Tong University, 1678 Dongfang Road, Shanghai 200127, China; majing@scmc.com.cn; 3 Department of Clinical Epidemiology and Biostatistics, Shanghai Children’s Medical Center, School of Medicine, Shanghai Jiao Tong University, 1678 Dongfang Road, Shanghai 200127, China

**Keywords:** congenital diarrhea, protein-losing enteropathy, *DGAT1*, mutation, RNA sequencing

## Abstract

**Background**: Congenital diarrhea is persistent diarrhea that manifests during the neonatal period. Mutations in *DGAT1*, which is crucial for triglyceride synthesis and lipid absorption in the small intestine, are causal factors for congenital diarrhea. In this study, we aimed to determine the value of tissue RNA sequencing (RNA-seq) for assisting with the clinical diagnosis of some genetic variants of uncertain significance. **Methods**: We clinically evaluated a patient with watery diarrhea, vomiting, severe malnutrition, and total parenteral nutrition dependence. Possible pathogenic variants were detected using whole-exome sequencing (WES). RNA-seq was utilized to explore the transcriptional alterations in *DGAT1* variants identified by WES with unknown clinical significance, according to the American College of Medical Genetics guidelines. Systemic examinations, including endoscopic and histopathological examinations of the intestinal mucosa, were conducted to rule out other potential diagnoses. **Results**: We successfully diagnosed a patient with congenital diarrhea and protein-losing enteropathy caused by a *DGAT1* mutation and reviewed the literature of 19 cases of children with *DGAT* defects. The missense mutation c.620A>G, p.Lys207Arg located in exon 15, and the intronic mutation c.1249-6T>G in *DGAT1* were identified by WES. RNA-seq revealed two aberrant splicing events in the *DGAT1* gene of the patient’s small intestinal tissue. Both variants lead to loss-of-function consequences and are classified as pathogenic variants of congenital diarrhea. **Conclusions**: Rare *DGAT1* variants were identified as pathogenic evidence of congenital diarrhea, and the detection of tissue-specific mRNA splicing and transcriptional effects can provide auxiliary evidence.

## 1. Introduction

Congenital diarrhea encompasses a heterogeneous spectrum of disorders characterized by persistent diarrhea that typically manifests during the neonatal period. Among the known genetic etiologies, mutations in the diacylglycerol O-acyltransferase 1 (*DGAT1*) gene have been identified as pivotal causal factors in the pathogenesis of various congenital diarrheal presentations [1,2]. *DGAT1* encodes a key enzyme responsible for triglyceride synthesis and lipid absorption within the small intestine, playing a critical role in maintaining intestinal epithelial integrity and functionality. Loss-of-function mutations in *DGAT1* can disrupt lipid metabolism, leading to impaired assimilation of dietary fats and subsequent malabsorptive diarrhea [3]. *DGAT1* mutations significantly contribute to the pathogenesis of congenital diarrhea by compromising lipid metabolism and intestinal absorption. A thorough understanding of the phenotypic spectrum and clinical outcomes associated with *DGAT1* mutations, combined with a comprehensive review of documented mutation cases, is essential for enhancing the management of affected patients. Furthermore, elucidating the molecular mechanisms underlying *DGAT1*-associated diarrhea is crucial for the development of targeted therapies and improving clinical outcomes.

When evaluating the pathogenicity of rare genetic variants, tissue-specific transcriptome analyses can provide compelling evidence of pathogenicity. Tissue RNA sequencing (RNA-seq) has emerged as a powerful tool for investigating transcriptional alterations associated with variants of uncertain significance (VUSs) that are predicted to affect mRNA splicing, enabling a more precise molecular diagnosis in cases where conventional genetic analysis remains inconclusive.

In this study, we aimed to assess the utility of RNA-seq for establishing the pathogenicity of *DGAT1* variants of uncertain clinical significance in a patient presenting with congenital diarrhea. Following an extensive clinical evaluation, whole-exome sequencing (WES) identified two previously uncharacterized *DGAT1* variants: a missense mutation and an intronic mutation. RNA-seq analysis performed on intestinal tissue obtained during endoscopy revealed aberrant splicing events, confirming the pathogenic consequences of these variants. Our findings not only validated the impact of these *DGAT1* mutations on gene expression but also characterized a previously unreported missense mutation associated with partial functional loss and a severe clinical phenotype. This study expands the phenotypic spectrum of *DGAT1*-associated congenital diarrhea and highlights the critical role of RNA-seq in elucidating the pathogenic effects of rare genetic variants.

## 2. Materials and Methods

### 2.1. Patient Evaluation

We conducted a comprehensive evaluation of a 4-month-old patient presenting with recurrent vomiting and diarrhea, encompassing both physical examinations and investigative procedures, in October 2023 at Shanghai Children’s Medical Center, with follow-up conducted until the patient reached 1 year of age. The physical examination included assessment of growth parameters and an abdominal examination. Laboratory investigations included stool studies, fecal culture, electrolyte analysis, complete blood count, blood lipid profile, immune function tests, organic acid analysis, and evaluation of serum albumin and total protein levels. Imaging studies consisted of abdominal ultrasonography, upper gastrointestinal series, and endoscopic and histopathological examinations of the intestinal mucosa. This comprehensive approach facilitated a detailed assessment of the patient, enabling a precise diagnosis and the development of tailored treatment strategies.

### 2.2. Whole-Exome Sequencing and Analysis

WES was conducted according to previously established protocols [4]. Genomic DNA was extracted from the patient’s peripheral blood and fragmented into fragments of 150–200 bp. Sequencing libraries were constructed using the SureSelect XT Human All Exon V6 kit (Agilent Technologies, Santa Clara, CA, USA), and sequencing was performed on an Illumina NovaSeq 6000 System (Illumina, San Diego, CA, USA). Data quality control and adapter sequence removal were performed using Fastqc (Babraham Research Institute, Cambridge, UK) and Fastp (Visible Genetics Inc., Toronto, ON, Canada) tools, respectively. Alignment with the reference genome was performed using SpeedSeq (Ira Hall Lab, St. Louis, MO, USA). The Genome Analysis Toolkit (Broad Institute, Cambridge, MA, USA) was employed to identify variations in the BAM file that met the quality control criteria, generating a VCF file. Variations in the VCF files were annotated using Ingenuity Variant Analysis (Ingenuity Systems, Redwood City, CA, USA) and Translational Genomics Expert platforms. All potential variants were confirmed by Sanger sequencing and validated using parental test results. Copy number variants were detected using CNVkit open-source software [5].

### 2.3. RNA Sequencing and Data Analysis

First, small intestinal tissue was obtained from the patient, and total RNA was extracted using RNeasy Kits (Qiagen, Germany). Poly(A) mRNA was then selected, and cDNA libraries were prepared using the Illumina TruSeq Stranded mRNA Library Prep kit (Agilent Technologies), following the manufacturer’s instructions. Next, the cDNA libraries were sequenced on an Illumina NovaSeq 6000 System (Illumina) using 150 bp paired-end reads.

The generated FASTQ files underwent quality control and adapter sequence removal using Fastqc v.0.11.9 (Babraham Research Institute) and Fastp v.0.20.1 (Visible Genetics, Inc.), respectively. Subsequently, to detect gene fusions and enhance the sensitivity of novel splice junction detection, clean data were aligned to the reference human genome (hg19) using STAR v.2.7.8a (Cold Spring Harbor, New York, NY, USA) with the parameters twopassMode and chimeric output function (chimSegmentMin = 12, twopassMode = ‘Basic’). Gene level quantifications were then performed using RSEM (v1.2.28). Next, differentially expressed genes were identified and compared to controls with an adjusted *p* value < 0.05, and cutoff (*q*-value) using DESeq2 (v.1.26.0). LeafCutter was employed to assess the statistical significance of the differences in the quantity of each splicing event (minclureads = 30; maxintronlen = 500,000; mincluratio = 1 × 10^−5^). Each patient was compared with all other controls for differential splicing analysis (min_samples_per_group = 1; min_samples_per_intron = 1). The resulting *P* values were corrected for multiple testing using a family-wise error rate approach. Finally, aberrant splicing events were visualized using Sashimi plots generated using MISO (v.0.5.4) and the Integrative Genomics Viewer. STAR-Fusion utilized chimeric and discordant read alignments identified by the STAR aligner to predict fusion events.

### 2.4. Literature Review of Reported DGAT1 Variant Cases

We conducted a systematic literature search across PubMed, Google Scholar, and genetic disease databases using keywords related to “*DGAT1*”, “congenital diarrhea”, and “gene mutation”. Studies published until December 2023 that reported cases of congenital diarrhea with confirmed *DGAT1* mutations were included.

## 3. Results

### 3.1. Clinical Diagnosis of Congenital Diarrhea

The patient was a male infant born at full term following spontaneous labor, with normal birth weight, length, and head circumference. He was the first child of a non-consanguine Chinese family and had no siblings. Vomiting began shortly after birth, accompanied by failure to thrive, prompting medical intervention. By 4 months of age, he required hospital admission due to watery diarrhea, hypotonia, severe malnutrition, and total parenteral nutrition (TPN) dependence.

**Figure 1:**
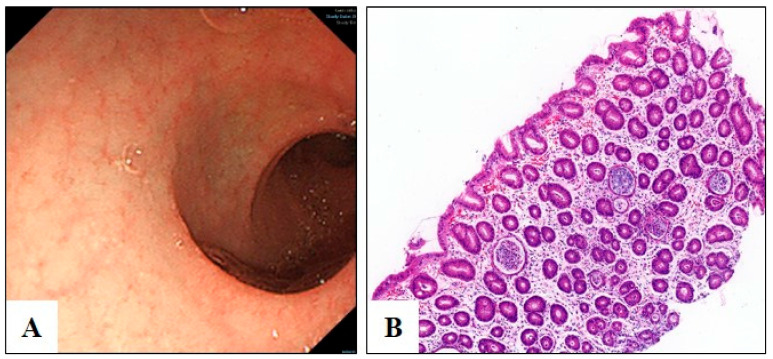
Endoscopic and pathological findings of the descending duodenum. (**A**) Duodenal descending endoscopic mucosa appears normal. (**B**) Hematoxylin-eosin staining showing no pathological lymphangiectasia, minimal villous atrophy, absence of crypt hyperplasia, and no evidence of lymphangiectasia.

Upon admission, his weight was 3.6 kg (birth weight: 3.1 kg), and he exhibited electrolyte imbalances, refractory hypoproteinemia, metabolic acidosis, and secondary hypothyroidism. Urine organic acid analysis revealed elevated levels of octamethylene and sebacic acids, suggesting enhanced fatty acid oxidation. Endoscopic examination showed normal mucosa, and biopsies from the duodenum and colon ruled out conditions such as small intestinal lymphangiectasia, food protein-induced colitis, microvillous inclusion disease, and tufting enteropathy (Figure 1).

Following genetic confirmation of the diagnosis, treatment was initiated with low-osmotic-pressure hydrolyzed milk powder enriched with medium-chain fatty acids (ALFARE formula), resulting in the resolution of diarrhea and discontinuation of TPN within 1 month. The patient was monitored for 6 months after discharge, with no recurrence of diarrhea or vomiting. However, weight gain remained slow, with a total increase of only 2 kg between 5 months and 1 year of age.

### 3.2. Identification of Genomic Variants in DGAT1 Gene

**Figure 2:**
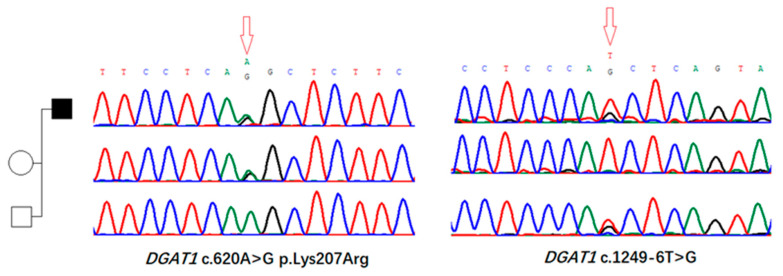
Exome sequencing revealed two mutations in *DGAT1*. A missense mutation c.620A>G, p.Lys207Arg located in exon 15 and an intronic mutation c.1249-6T>G in intron 7. Subsequent Sanger sequencing of the proband and his parents reveal the compound heterozygote state of mutations in the proband.

Exome sequencing revealed two mutations in *DGAT1*: the missense mutation c.620A>G, p.Lys207Arg located in exon 15, and the intronic mutation c.1249-6T>G in intron 7. Sanger sequencing of the proband and his parents revealed a compound heterozygous state of mutations in the proband (Figure 2). The intronic mutation c.1249-6T>G was absent from GnomAD v.4.1.0, while the missense mutation c.620A>G is seen only once in over 1,610,000 alleles in the GnomAD v.4.1.0 database. According to the American College of Medical Genetics (ACMG) guidelines for interpretation of genetic variants, both mutations were categorized as “unknown clinical significance”. However, the results of SpliceAI software predicting the impact of the missense mutation and intronic variant carried by the patient on mRNA splicing suggested a high degree of deleteriousness (SpliceAI score 0.9 for c.620A>G and 1.0 for c.1249-6T>G). Thus, further pathogenic evidence was required for diagnosis.

### 3.3. Aberrant Splicing Event Revealed by RNA Sequencing

**Figure 3:**
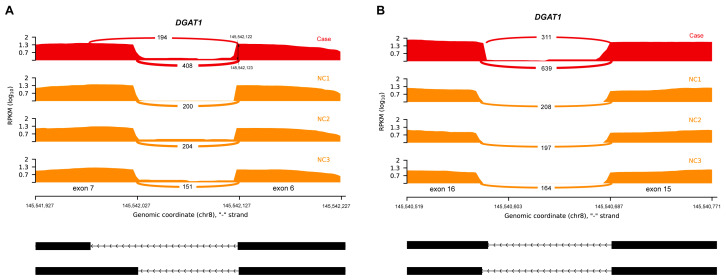
Sashimi plot of aberrant splicing events in *DGAT1*. (**A**) Exonic splice gain caused by an A>G donor splice site, creating a variant in the patient (red). The 3′ss of this aberrant transcript exhibited a shift, with one nucleotide upstream compared to the normal 3′ss (orange). (**B**) Exon extension caused by a donor c.1249-6T>G extended splice site variant.

We identified two aberrant splicing events in the *DGAT1* gene of the patient, resulting in an exonic splice gain and exon extension (Figure 3). The former was caused by the NM_012079.6:c.620A>G mutation, leading to the emergence of a new 5′ splice site (5′ss) on exon 7 of *DGAT1*. This aberrant splicing event also resulted in a change in the 3′ splice site (3′ss) on exon 6, with the chromosomal position shifting from 15,542,123 to 15,542,122. Both events led to a reading-frame shift of the coding protein, and it is presumed that the abnormal lower transcript count is due to the presence of the nonsense-mediated decay mechanism.

### 3.4. Review of Reported DGAT1 Cases

This study summarized a cohort with (likely) pathogenic genomic variants in *DGAT1*, all presenting with a phenotype of vomiting, congenital diarrhea, failure to thrive, and malnutrition (Table 1). As of December 2023, nine articles reported 19 cases of children with *DGAT1* gene defects, revealing 21 types of mutations in *DGAT1*, including missense, nonsense, and insertion–deletion mutations. Among them, splice site mutations were the most common, with 10 pathogenic splice site mutations reported [2,6,7,8,9,10,11,12,13,14].

## 4. Discussion

*DGAT1* encodes a key enzyme involved in triglyceride biosynthesis and lipid absorption, which is essential for maintaining intestinal homeostasis. Loss-of-function mutations in *DGAT1* result in protein-losing enteropathy (PLE), characterized by early-onset, non-bloody watery diarrhea, with patients often presenting with severe dehydration, malnutrition, and growth retardation, particularly during infancy [13]. These cases typically exhibit poor responses to conventional treatments, making early genetic diagnosis and personalized intervention essential for improving clinical outcomes. Although histopathological findings in *DGAT1*-related diarrhea remain less well characterized compared to other hereditary diarrheal disorders, abnormalities in lipid metabolism and mucosal structural alterations have been implicated [8]. Some affected patients may also develop extraintestinal symptoms, such as dry skin and nutritional anemia [13].

To date, 19 cases of *DGAT1*-related congenital diarrhea have been reported, with 21 distinct mutations identified. The majority of these mutations result in loss-of-function consequences, either due to protein truncation or impaired enzymatic activity, ultimately leading to severe diarrhea and failure to thrive [13]. Previous studies have demonstrated a significant reduction in *DGAT1* mRNA expression in fibroblasts obtained from affected individuals, indicating that missense mutations can also result in loss-of-function effects [11].

Different mutations in *DGAT1* can lead to varying degrees of disease severity, ranging from complete loss of enzymatic function to partial functional impairment, which may influence the clinical presentation and response to treatment [6]. Early identification of *DGAT1* mutations through genetic testing enables timely initiation of personalized management strategies, including dietary modifications and nutritional support, which can significantly improve patient outcomes.

A limitation of our study is the lack of direct functional validation of *DGAT1* protein activity. However, by performing RNA-seq analysis of intestinal tissue, we were able to provide compelling evidence of the pathogenicity of the identified variants through the detection of aberrant splicing events. For other rare diseases where tissue-specific RNA expression is not easily accessible, this approach may not be feasible, underscoring the need for alternative strategies to assess the functional consequences of genetic variants.

RNA-seq has emerged as a valuable tool in molecular diagnostics, enabling the detection of transcriptomic abnormalities, including aberrant splicing, differential gene expression, and monoallelic gene expression. It has been reported to enhance diagnostic yield by 7.5–36% in cases where DNA sequencing alone is insufficient to establish a definitive diagnosis [15]. However, a major challenge in the clinical application of RNA-seq lies in the tissue specificity of gene expression. Obtaining sufficient expression of disease-relevant genes in clinically accessible tissues such as blood or fibroblasts may be challenging, especially for disorders affecting tissues with low or tissue-specific gene expression profiles. For example, in many Mendelian disorders involving intellectual and developmental disabilities, disease-associated genes may be exclusively expressed in the brain, limiting their detection in peripheral tissues.

In our study, we addressed this limitation by obtaining intestinal mucosal tissue during endoscopy to ensure adequate representation of *DGAT1* expression for transcriptomic analysis. Due to insufficient expression of some genes in peripheral blood, for surgical patients, retaining diseased tissue for testing, or testing through fibroblasts provides a higher likelihood of accurate molecular diagnoses by identifying disease-relevant transcriptomic changes.

In our study, WES identified two previously uncharacterized *DGAT1* variants—a missense mutation (c.620A>G, p.Lys207Arg) in exon 15 and an intronic mutation (c.1249-6T>G)—that were initially classified as VUSs according to the ACMG variant classification guidelines. However, considering the strong phenotypic concordance between the patient’s clinical presentation and *DGAT1*-associated congenital diarrhea, these variants were suspected to be potential genetic etiologies.

Bioinformatic analysis using Splice AI suggested a possible impact of these variants on mRNA splicing, prompting the use of RNA-seq for functional validation. RNA-seq analysis of intestinal tissue samples revealed aberrant splicing events resulting from both variants, ultimately leading to loss-of-function consequences. This approach allowed us to refine the classification of these variants as pathogenic and confirmed their direct impact on *DGAT1* gene expression.

Currently, no targeted therapies are available to restore *DGAT1* activity in affected patients. However, tailored dietary interventions have shown promising results in alleviating symptoms and preventing complications. Most patients benefit from low-fat or fat-free diets supplemented with medium-chain triglycerides (MCTs), which bypass the conventional lipid absorption pathway. Among the 19 reported cases, one child died from complications at 17 months, whereas others who received appropriate dietary interventions experienced resolution of gastrointestinal symptoms and were able to discontinue intravenous nutrition.

Tailored dietary recommendations may be required based on the nature of the *DGAT1* mutation. For individuals with complete loss of *DGAT1* function, it is recommended that fat provides only 4–7% of total energy intake due to its low energy density, while those with partial loss of function may tolerate higher fat intake, up to 10% of total energy. Supplementation with oils such as canola and sunflower oils is often recommended for patients adhering to a fat-free diet. In one case, a patient receiving an extensively hydrolyzed MCT formula (40%) experienced improvement in diarrhea but demonstrated insufficient weight gain, with growth parameters remaining below the third percentile. This underscores the importance of long-term growth monitoring and individualized nutritional management in patients with *DGAT1* deficiency.

In conclusion, in a rare case of a child presenting with watery stools, decreased muscle tone, and severe malnutrition, a missense and intronic site mutation in *DGAT1* was identified through routine WES. Further investigation using RNA sequencing specifically targeting intestinal tissue confirmed that both mutations affected the mRNA splicing of *DGAT1*. The discovery and diagnosis in this case suggest that in the process of determining the pathogenicity of rare variants, the detection of tissue-specific mRNA splicing, and transcriptional effects can provide auxiliary evidence. Our research and case report also update the mutation spectrum of *DGAT1*, providing additional information on genotype–phenotype correlations and the underlying pathogenic mechanisms, thereby offering a basis for the diagnosis and treatment of related hereditary diarrheal diseases. Mutations in *DGAT1* represent pathogenic evidence for congenital diarrhea. The detection of tissue-specific mRNA splicing and transcriptional effects within the intestinal mucosa provides supplementary evidence for clinical diagnosis. Therefore, tissue-specific mRNA analysis can complement next-generation sequencing in the diagnosis and management of related genetic disorders, particularly for variants of uncertain clinical significance.

## Figures and Tables

**Table 1:** *DGAT1* mutations identified for congenital diarrhea.

Patient No.	Variants	Phenotype	Onset Age	Reference
1–2	c.895-1G>A;	vomiting, PLE, malnutrition, watery diarrhea, hypertriglyceridemia	N/A	Ye et al., 2019 [7]
3	c.1249-6T>G	early-onset PLE, CDD, malnutrition, hypoalbuminemia, lymphopenia, edema	N/A	Ye et al., 2019 [7]
4	c.895-1G>A;c.751+1G>C	vomiting, PLE, malnutrition, watery diarrhea, hypertriglyceridemia	8 months	Xu et al., 2020 [6]
5–6	c.314T>C (p.Leu105Pro)	early-onset vomiting and/or diarrhea, hypoalbuminemia, PLE	19 months/ 26 months	van Rijn et al., 2018 [8]; Gluchowski et al., 2017 [9]
7	c.1202G>A (p.W401*)	failure to thrive, vomiting, diarrhea, hypoalbuminemia, hypogammaglobulinemia, edema	5 months	van Rijn et al., 2018 [8]
8	c.573_574delinsCCCATCCCCCCTCGCCCATCT, p.Val192Profs*99)	failure to thrive, vomiting, diarrhea, hypoalbuminemia, hypogammaglobulinemia, edema	11 months	van Rijn et al., 2018 [8]
9	c.937-1G>A	failure to thrive, vomiting, diarrhea, hypoalbuminemia, hypogammaglobulinemia	8 years	van Rijn et al., 2018 [8]
10	c.953insG (p.Ile319Hisfs*33)	failure to thrive, vomiting, bloody and watery diarrhea, hypoalbuminemia, hypogammaglobulinemia	2 years	van Rijn et al., 2018 [8]
11	c.629_631delCCT (p.Ser210del)	severe vomiting, diarrhea	20 months	van Rijn et al., 2018 [8]
12	c.751+2T>C	vomiting, CDD, PLE, malnutrition, electrolyte abnormalities, TPN dependence	7 weeks	Schlegel et al., 2018 [10]
13	c.884T>C (p.Leu295Pro)	vomiting, CDD, PLE, malnutrition	2 months	Stephen et al., 2016 [11]
14	c.629_631delCCT (p.Ser210del)	vomiting, diarrhea, severe malnutrition, hypotonia	2 weeks	Gupta et al., 2020 [12]
15	c.676+1G>A	vomiting, diarrhea, vitamin D deficiency	N/A	Gupta et al., 2020 [12]
16	c1013_1015delTCT (p.Phe338del)	severe diarrhea, PLE, fat-soluble vitamin deficiency, secondary hyperparathyroidism	2 months	Ratchford et al., 2017 [13]
	c.1260c>G (p.Ser420Arg)			
17	c.1310A>G (p.Gln437Arg)	vomiting, growth failure, vitamin D deficiency	N/A	Gupta et al., 2020 [12]
	c.981+1G>T			
18	c.1311+1G>A	vomiting, growth failure, hypogammaglobulinemia, TPN dependence	N/A	Gupta et al., 2020 [12]
	c.1462delG (p.Ala488Profs*226)			
19	c.676+1G>T	vomiting, diarrhea, malnutrition	1 month	Chen et al., 2020 [14]
	c.367_368delCT			
Our case	c.620A>G, p.Lys207Arg	vomiting, watery diarrhea, hypotonia, severe malnutrition, TPN dependence	4 months	
	c.1249-6T>G			

## Data Availability

The original contributions presented in this study are included in the article. Further inquiries can be directed to the corresponding author.
